# Cost Burden of End of Life Care Across Gradients of Cognitive Impairment Among Nursing Home Residents with Cancer

**DOI:** 10.1093/geroni/igaf122.2605

**Published:** 2025-12-31

**Authors:** Long Vu, Siran Koroukian, Johnie Rose, David Warner, Sara Douglas, Nicholas Schiltz

**Affiliations:** Case Western Reserve University School of Medicine, Cleveland, Ohio, United States; Case Western Reserve University School of Medicine, Cleveland, Ohio, United States; Case Western Reserve University School of Medicine, Cleveland, Ohio, United States; The University of Alabama at Birmingham, Birmingham, Alabama, United States; Case Western Reserve University, Cleveland, Ohio, United States; Case Western Reserve University, Cleveland, Ohio, United States

## Abstract

Compared to aggressive end of life (EOL) care, palliative and hospice care are associated with cost savings in older adults with cancer. It is not known, however, whether these savings generalize to nursing home (NH) residents, who may present with varying levels of cognitive impairment (COG-I) and complex multimorbidity. Using the 2013-2017 Surveillance, Epidemiology, and End Results Registry linked with the Minimum Data Set and Medicare claims, we identified 40,833 older NH residents who died with cancer. We examined the total costs of all Medicare payments within the last 30 days of life across gradients of COG-I, and between markers of aggressive EOL care received. 49.2% of identified NH residents were cognitively intact; 24.4% had mild COG-I; 19.7% had moderate COG-I; and 6.7% had severe COG-I. Adjusting for inflation and other clinical variables, compared to those cognitively intact, average total costs were $682 (95% confidence interval [CI]: $11-$1,353) and $1,279 (95% CI: $561-$1,997) lower for those with mild and moderate COG-I, respectively. Costs for those with severe COG-I did not differ. Any aggressive EOL care received increased average costs across all COG-I levels by $25,142 (95 % CI: $24,338-$25,946). Conversely, each additional day enrolled in hospice yielded an average savings of $546 (95% CI: $517-$577). Our results reinforce the high cost of aggressive EOL care and the savings benefit of hospice care in older NH residents with cancer. Although costs were slightly lower at increasing levels of COG-I, the most severely impaired had costs similar to their non-impaired counterparts.

